# Supplementary material for: β-Ketoenamine Covalent Organic Frameworks—Effects of Functionalization on Pollutant Adsorption
Source: Polymers (Basel). 2022 Jul 29;14(15):3096. doi: 10.3390/polym14153096 (PMC9370968; doi:10.3390/polym14153096)
Supplement: Supplementary file 1 [file polymers-14-03096-s001.zip › polymers-1798978-supplementary.pdf]

polymers MDPI

Supplementary Information

β-Ketoenamine Covalent Organic Frameworks – Effects of Functionalization on Pollutant Adsorption

Tiago F. Machado, Filipa A. Santos, Rui F.P. Pereira, Verónica de Zea Bermudez, Artur J.M. Valente*, M. Elisa Silva Serra and Dina Murtinho

S1. Adsorption Experiments

Table S1. Statistical parameters for UV-Vis quantification of MB and MO adsorbates.

Adsorbate	λ_{max} (nm)	b1	b0	R^2
MB	664	0.158 ± 0.003	0.01 ± 0.01	0.9983
MO	463	0.0770 ± 0.0001	-0.0080 ± 0.0006	0.9999
Cu(II)	325	0.0200 ± 0.0002	0.014 ± 0.007	0.9991
Ni(II)	232	0.0071 ± 0.0005	0.09 ± 0.03	0.9741
Pb(II)	217	0.0049 ± 0.0002	0.03 ± 0.02	0.9897
Cd(II)	228	0.028 ± 0.002	0.050 ± 0.03	0.9706

S1. Characterization

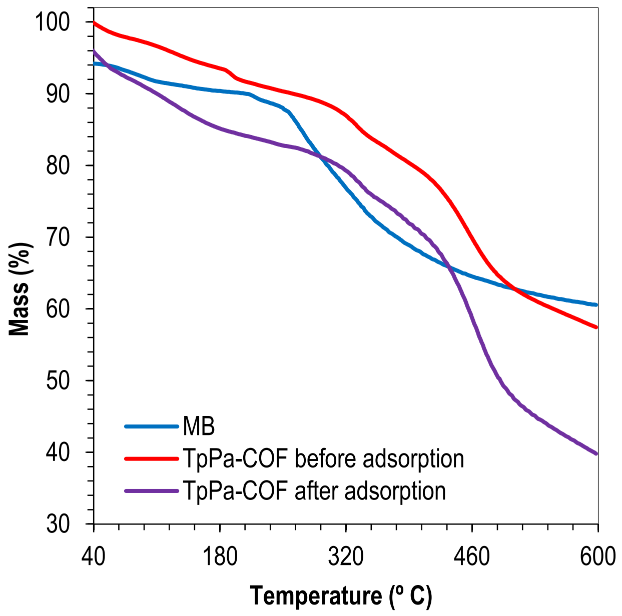

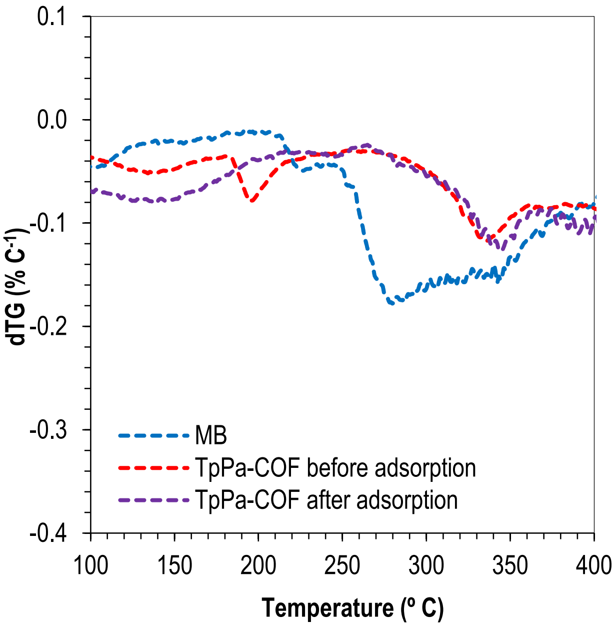

Figure S1. Thermograms (solid) and dTG (dashed) of MB (blue) and TpPa-COF before (red) and after (purple) MB adsorption.

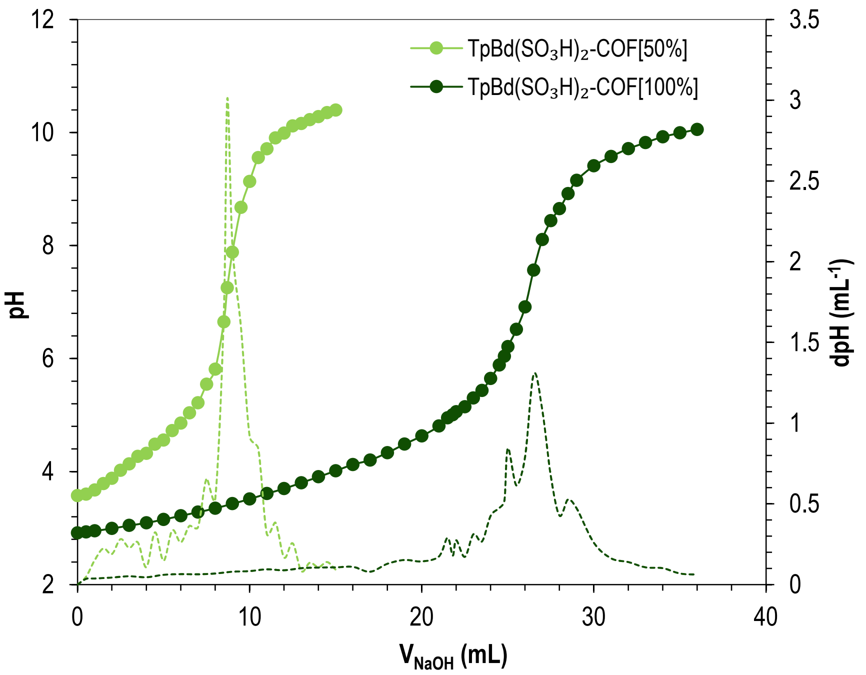

Figure S2. pH (solid) and dpH (dashed) during the titration of TpBd(SO_3H)$_2$-COF[50%] (light green) and TpBd(SO_3H)$_2$-COF[100%] (dark green).
